# Fertility Outcomes following Laparoscopy-Assisted Hysteroscopic Fallopian Tube Cannulation: A Preliminary Study

**DOI:** 10.1155/2018/7060459

**Published:** 2018-06-06

**Authors:** Joseph I. Ikechebelu, George U. Eleje, Prashant Bhamare, Ngozi N. Joe-Ikechebelu, Chidimma D. Okafor, Abdulhakeem O. Akintobi

**Affiliations:** ^1^Life Institute for Endoscopy, Life Specialist Hospital Limited, 7 Ikemba Drive, Umudim, Nnewi, Anambra State, Nigeria; ^2^Effective Care Research Unit, Department of Obstetrics and Gynaecology, Faculty of Medicine, Nnamdi Azikiwe University, Nnewi Campus, Nnewi, Nigeria; ^3^Department of Obstetrics and Gynaecology, Nnamdi Azikiwe University Teaching Hospital, Nnewi, Anambra State, Nigeria; ^4^Matushree Hospital, Charkop, Kandivali, Mumbai, India; ^5^Department of Community Medicine, Chukwuemeka Odumegwu Ojukwu University, Amaku, Awka, Nigeria; ^6^Department of Obstetrics and Gynaecology, Asokoro District Hospital, FCT Abuja, Nigeria

## Abstract

**Objectives:**

To determine fertility outcomes following laparoscopy-guided hysteroscopic tubal cannulation for cornual obstruction.

**Study Design:**

A prospective cohort study in Life Institute for Endoscopy at Life Specialist Hospital Nnewi, Southeast Nigeria. Patients with unilateral or bilateral cornual tubal obstruction as the only cause of infertility were included. Outcome measures included successful tubal recanalization, procedural complications, conception rates (first spontaneous conception after the procedure), and live birth rates.

**Results:**

Forty-nine infertile women were assessed for eligibility, but 27 met the inclusion criteria. Of 27 women, 24 (88.9%) had bilateral cornual obstruction and 3 (11.1%) had unilateral obstruction. Only three (11.1%) patients had failed cannulation. Successful recanalization rate was 90.2% (46/51) per tube and 88.9% (24/27) per patient. In the 24 patients with successfully recanalization, six spontaneous pregnancies (25.0%) and two intrauterine insemination-assisted pregnancies (8.3%) occurred within first six months of follow-up. All the eight (100.0%) pregnancies were intrauterine. The overall conception rate and live birth rate was 33.3%. There were no pre- or postprocedural complications.

**Conclusion:**

Successful recanalization rate was 90.2% per tube and 88.9% per patient with a conception rate of 33.3%. Women with only cornual obstruction should be considered first for laparoscopy-assisted hysteroscopic cannulation before assisted reproduction.

## 1. Introduction

Infertility remains a multifaceted condition with substantial medical, economic, and psychosocial influence in our environment. Quite often, a number of women distressed from infertility are bewildered with the fact that the solitary reason for their inability to conceive is tubal in origin [1, 2]. Since different tubal sites could be affected, hysterosalpingography (HSG) remains a valuable modality employed at the commencement of infertility evaluation in order to identify the actual site of tubal affectation.

The findings of cornual obstruction on hysterosalpingogram can be due to several reasons including tubal spasm, mucus plugs, debris, or true cornual blockage [1, 3]. In order to distinguish between true cornual obstruction and other causes, several methods including saline sonohysterosalpingography and laparoscopy and dye test have been advocated. While laparoscopy and dye test assess tubal patency, other pelvic, peritoneal, and tubal pathologies that can affect management outcomes are also assessed [4].

Compared to tubal surgery, laparoscopy-assisted hysteroscopic tubal cannulation is an innovative method which is valuable for the management of infertile women with cornual obstruction. With the aid of laparoscopy, it becomes easier to achieve tubal cannulation and examination of the pelvis in its entirety via the hysteroscopic approach. This also ensures that supplementary problems of the fallopian tubes such as adhesions and endometriosis are identified and addressed adequately [5].

Previous studies have revealed a recanalization success rate of 76% and intrauterine pregnancy rate of 39% following laparoscopy-assisted hysteroscopic tubal cannulation [3, 6, 7]. Previous published studies have also documented the procedure to be safe for both diagnostic and therapeutic purposes [1, 3]. Thus, tubal cannulation remains a valuable approach in the management of couples with tubal infertility and could selectively be offered to women prior to in vitro fertilization (IVF). Although tubal surgery (resection and anastomoses) is often becoming an unpopular procedure for management of infertility owning to poor results, IVF remains the most reliable treatment options for women with proximal tubal occlusion (PTO) in Nigeria. Despite its high demand, IVF uptakes are hampered by a number of factors such as religion, cost, and success rates. Laparoscopy-assisted hysteroscopic tubal cannulation has been invented to circumvent these problems inherent in assisted reproductive procedures.

To the best of our knowledge, we could not find any published study in Nigeria on laparoscopy-assisted hysteroscopic tubal cannulation. We planned this pilot study to determine the fertility outcomes after laparoscopy-assisted hysteroscopic tubal cannulation.

## 2. Patients and Methods

This was a prospective cohort study evaluating all infertile women with cornual tubal occlusion who underwent laparoscopic-hysteroscopic tubal cannulation in Life Institute for Endoscopy at Life Specialist Hospital Nnewi, Southeast Nigeria, a private training and research facility, from April 1, 2013, to August 30, 2015. The study protocol was approved by the Nnamdi Azikiwe University Teaching Hospital Ethics Committee. All women voluntarily agreed to participate in the study and gave their written informed consent. The procedure was performed as a day case.

Tubal cannulation sets used have the following: (a) an outer catheter, the end of which was impinged against the tubal ostia for the selective dye test during hysteroscopy. The selective dye test aimed to exclude false positive outcomes from HSG or laparoscopy and the dye test due to preferential dye flow through one tube; (b) an inner catheter for tubal cannulation or catheterisation, which was passed with the guide wire advanced flush with its tip. This combination was passed into the proximal 1-2 cm after tube has been confirmed blocked by laparoscopic or hysteroscopic dye test; and (c) an innermost guide wire which provided some rigidity to the inner tube to minimize kinking and was pushed carefully beyond the tip of the inner tube for proximal tubal recanalisation only after inner tube cannulation, and dye test persistently show blockage. An operative hysteroscopy sheath with size-7 channel was used. They were followed up for at least six months, and pregnancy during this period was documented. For the purpose of analysis, only the first conception was taken into account.

### 2.1. Exclusion Criteria

Women with active genital infection or tuberculosis were excluded from the study. Women with obvious disease in the tubes (hydrosalpinx, adhesions, distorted tubal structure due to multiple uterine myomas, and distal tubal blockage) were also excluded.

### 2.2. Patient Selection and Procedure

In our center, diagnostic laparoscopy and dye test under general anaesthesia were used to assess the tuboperitoneal status of the infertile women included in the study. At diagnostic laparoscopy, endometriosis and pelvic adhesions were evaluated, and therapeutic surgery, including cauterisation and adhesiolysis, was offered if indicated. Tubal patency was tested using methylene blue dye injected via uterine cannular fixed to the cervical canal. The diagnosis of cornual occlusion was made if there is no passage of dye to the fallopian tubes. In women with cornual occlusion who meet the inclusion criteria, hysteroscopic tubal cannulation was performed under laparoscopy guidance to restore the tubal patency. An operative hysteroscope (OM Surgicals, Mumbai, India) was introduced into the uterine cavity with normal saline as distending medium and the tubal ostium identified. A tubal cannulation catheter (OM Surgicals, Mumbai, India) consisting of a 50 cm long Teflon catheter and a steel guide wire of 0.018 mm in diameter were then introduced via the operating channel into the ostium to enter the cornual segment to about 1 cm. Once the cornual segment was cannulated with the guide wire, the latter was withdrawn and diluted methylene blue dye was injected directly through the Teflon catheter. The success of the recanalisation was assessed with evidence of spillage of the dye into the peritoneal cavity.

Laparoscopy was used for both monitoring the procedure and assessing the success of recanalisation. Prophylactic antibiotic was given for the procedure. Demographical data and operative details, including tubal patency after the hysteroscopic tubal cannulation, reproductive outcome, and complications (like infection, tubal or uterine perforations), were evaluated and documented.

All women were followed up via clinic appointment or telephone consultation for those from distant destinations. Proforma was employed which included questions on duration of infertility, time interval to spontaneous conception after the tubal cannulation, interval before seeking assisted reproductive methods, the time and mode of conception, and their pregnancy outcome (i.e., whether it was an ectopic pregnancy, miscarriage, or live birth).

Primary outcome measures were the success rate of recanalization (success rate per woman and per tube) and the pregnancy rate after the procedure. Secondary outcome measures included pregnancy outcome, number, and type of intra- and postoperative complications, rate of ectopic pregnancy, multiple pregnancies, and cost savings from avoiding IVF treatment.

No formal sample size calculations were made because of the pilot nature of the study. All Statistical analyses were performed via epi info 2008 version 3.5.1 (Centers for Disease Control and Prevention, Atlanta, GA) and Stata PASS version 10.0 (NCSS; Kaysville, Utah, USA). Univariate analysis was done using the Fisher exact test to compare categorical variables. The data are expressed as mean ± SD or number (percentage). Risk ratios (RRs) and 95% confidence intervals (CIs) were also calculated. A *p* value of <0.05 was considered statistically significant.

## 3. Results

**Figure 1:**
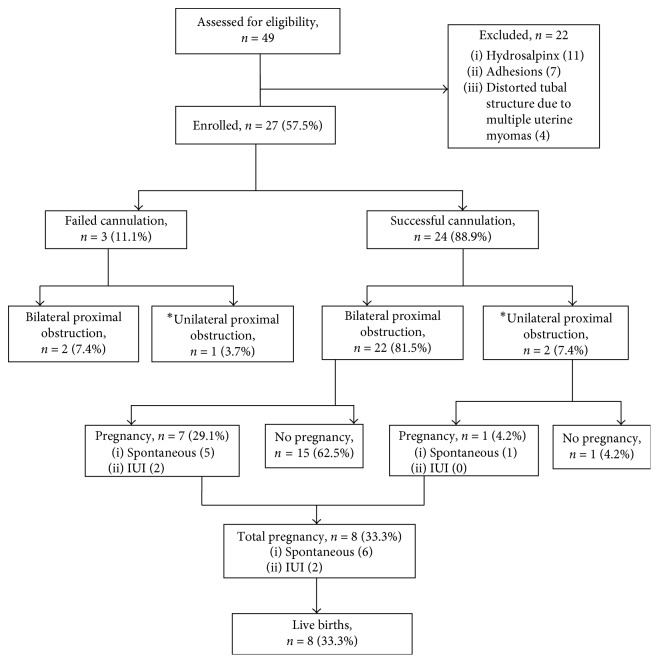
Flow pattern of women that had proximal tubal cannulation. IUI = intrauterine insemination. ^*∗*^Previous unilateral salpingectomy.

During the study period, a total of 49 infertile women were assessed for eligibility, but 27 cases met the inclusion criteria and were enrolled. The flow pattern is shown in Figure 1. The mean age was 35.7 ± 4.6 years, and the mean duration of infertility was 7.8 ± 3.7 years. Up to 13 (48.1%) of the women had infertility for 5 years or below while 14 (51.9%) has had infertility for greater than 5 years.

Table 1 shows the relationship between age, type of infertility, and pregnancy outcome. There was no statistically significant difference between age of patients and infertility types with the pregnancy outcomes (*p* > 0.05).

Ten (37.0%) women had primary infertility while 17 (63.0%) had secondary, giving a ratio of 1 : 2. Of the 27 women enrolled, 24 (88.9%) had bilateral proximal obstruction and 3 (11.1%) had unilateral obstruction (they had previous unilateral salpingectomy). Twenty-four women had successful recanalization given a rate of 88.9% (24/27) per woman and 90.2% (46/51) per tube. Only three (11.1%) women had failed cannulation, two of which failed in both obstructed tubes and one in unilateral obstructed tube. These were due to long segmental obstruction. There was no recorded complication from the procedure.

In the 24 successful women, six spontaneous pregnancies (25.0%) and two intrauterine insemination-assisted pregnancies (8.3%) occurred within the first twelve months of follow-up. All the eight pregnancies were intrauterine and ended in live births. The overall conception rate was 33.3% and live births rate of 33.3%. All the pregnancies were singleton and occurred in women with previous bilateral tubal obstruction. Of the 8 pregnancies, 4/10 (40.0%) were in women with primary infertility while 4/17 (23.5%) in women with secondary infertility.

The cost of tubal cannulation was in the average of $750 (US), with the range of $500–1000 (US). The cost of In vitro Fertilization and Embryo Transfer in our practice area is average of $3250 with a range of $1500–5000 (US). This is a savings of $2500 (US) for performing a tubal cannulation.

## 4. Discussion

This study has revealed that successful recanalization rate was 90.2% (46/51) per tube and 88.9% (24/27) per patient following laparoscopy-assisted hysteroscopic tubal cannulation in developing country settings. However, in the 24 patients with successfully recanalization, six spontaneous pregnancies (25.0%) and two intrauterine insemination-assisted pregnancies (8.3%) occurred within first six months of follow-up with all the eight (100.0%) pregnancies being intrauterine. The overall conception rate and live birth rate was 33.3% with no accompanying pre- or postprocedural complications.

In our practice, women with tubal obstruction diagnosed at HSG are often referred for diagnostic laparoscopy and dye test for accurate diagnosis of tubal obstruction [4]. Following this, laparoscopic-guided hysteroscopic tubal cannulation is performed if the woman meets the selection criteria. One advantage of tubal cannulation is that it does not involve a laparotomy procedure and its attendant risks [8]. As in our case, cornual tubal cannulation has been successfully performed with catheters, flexible atraumatic guide wires, and balloon systems under the guidance of ultrasound, endoscopy, or fluoroscopy [9–11]. Currently, the evidence available is uncertain in terms of the superiority of one method over the other [12, 13], but the use of atraumatic guide wires in our case has shown to be efficacious.

In our study, no case of uterine perforation was recorded. This finding is similar to prior report by Deaton et al. [3] which concluded that laparoscopic-hysteroscopic tubal cannulation is a safe procedure. Our study had a successful tubal cannulation rate of 88.9% per woman and 90.2% per tube. The overall pregnancy rate was 33.3% after a single tubal cannulation procedure, which is also comparable with previous studies that involved falloposcopy-guided tubal cannulation and/or IVF and embryo transfer seen in most centers [14, 15]. IVF on the other hand can only be performed in centers with cutting-edge facilities and vastly trained experts. Furthermore, a number of physical and psychological problems are linked with IVF, and the procedure may need to be repeated to accomplish the anticipated success rate [16].

With tubal recanalization, the challenge of multiple pregnancies and ovarian hyperstimulation syndrome may be averted, and unassisted pregnancies following one episode of successful recannalization are possible [1, 10, 12]. Tubal recanalisation could also be a substitute for infertile couples who forbid IVF due to ethical, religious, or economic reasons. Currently, the trend is moving from tubal reconstructive surgery to IVF-ET procedures in order to proffer solution to tubal infertility. As demonstrated in our study, tubal cannulation permits women opportunity to attain spontaneous pregnancy without need for IVF-ET in cases of tubal infertility. This study has become very crucial as it addresses a very important health issue, particularly in a part of the world where IVF is, far too quickly, offered for tubal factor infertility.

A very important consideration in our low-income country is the huge cost of IVF, with 30–45% recorded success per attempt unlike the hysteroscopic tubal cannulation which when performed as a day-case technique was demonstrated to be cost-effective. According to other studies, the postprocedure pregnancy rates vary from 20 to 55% [1, 7, 10, 12, 13, 17, 18], which is similar to that obtained with IVF procedure. In cases with coincidental distal tubal disease which is not amenable to surgery, laparoscopic-guided hysteroscopic tubal cannulation is not a suitable technique. Our cost analysis reveals an average savings of $2500 (US) for performing a tubal cannulation relative to a single IVF and ET. However, successful cannulation will still have the benefit of long-term savings; the treated tube may remain patent for more than one pregnancy. More recent less-invasive office procedure involving hysteroscopically-assisted transvaginal ultrasound tubal catheterization has been described [18].

In our study, we could not decipher any factor attributable to the huge success recorded following successful laparoscopically-assisted hysteroscopic tubal cannulation or successful pregnancy in women with bilateral tubal obstruction prior to the cannulation. Additionally, women with prior unilateral salpingectomy had no successful pregnancy even with successful recanalization. It appears these women with prior unilateral salpingectomy had more pathology in the tubes besides the occlusion. This is expected as tubal damage is the key factor responsible for tubal pregnancy ab initio. Since six women conceived spontaneously following the cannulation, and two required intrauterine insemination due to suboptimal semen parameters, clinicians should therefore be aware of this possibility and explore all these options before referral for IVF.

The present study has some limitations. The study is limited by short duration of follow-up and small study size as this is a preliminary study. Additionally, our study could not describe the history of chlamydia infection, degree of adhesion, semen parameters, and data of ovarian reserves, such as basal follicle stimulating hormone and antimularian hormone, which we could have been included in the results if available. Also, we could not determine the predictive factor of tubal damage in this study. Nevertheless, this is the first study in Nigeria, which examined the role of laparoscopy-assisted hysteroscopic proximal tubal cannulation in cases of exclusive tubal (cornual) factor infertility.

In conclusion, the results of this pilot study suggest that successful recanalization rate was 90.2% per tube and 88.9% per patient with a conception rate of 33.3%. We therefore confirm that laparoscopy-assisted hysteroscopic tubal cannulation is an effective procedure with minimal or no complication for infertile women with cornual tubal occlusion as the sole pathology. It should be considered as a first treatment in the selected group before referral for IVF and embryo transfer, and it is a suitable and appropriate substitute for couples with tubal infertility who cannot afford or consent to IVF.

## 5. Synopsis

There is remarkable success in tubal recanalization and pregnancy rates following the first study of laparoscopy-assisted hysteroscopic tubal cannulation in Nigeria.

## Figures and Tables

**Table 1:** Relationship between age, infertility type, and pregnancy outcome.

Age	Frequency	Infertility type		Pregnancy success		RR (95% CI)	*p* value
		Primary	Secondary	Pregnant	Nonpregnant		
25–29	1	0	1	1	0	—	—
30–34	10	5	5	4	6	0.40 (0.005–39.2)	0.542
35–39	11	3	8	3	8	0.27 (0.003–28.5)	0.450
40–44	5	2	3	0	5	U (0.0–15.6)	0.286
Total	27	10	17	8	19	—	—

RR = Risk ratio; 95% CI = 95% confidence interval; U = undefined.

## Data Availability

The datasets used and/or analyzed during the current study are available from the corresponding author on reasonable request.

## Abbreviations

HSG: Hysterosalpingography
US: United States
IVF: In vitro fertilization
PTO: Proximal tubal occlusion
IVF-ET: In vitro fertilization and embryo transfer.
